# Overriding native cell coordination enhances external programming of collective cell migration

**DOI:** 10.1073/pnas.2101352118

**Published:** 2021-07-16

**Authors:** Gawoon Shim, Danelle Devenport, Daniel J. Cohen

**Affiliations:** ^a^Department of Mechanical and Aerospace Engineering, Princeton University, Princeton, NJ 08540;; ^b^Department of Molecular Biology, Princeton University, Princeton, NJ 08540

**Keywords:** collective cell migration, electrotaxis, coordinated motion, E-cadherin, cell–cell adhesion

## Abstract

As collective cell migration is essential in biological processes spanning development, healing, and cancer progression, methods to externally program cell migration are of great value. However, problems can arise if the external commands compete with strong, preexisting collective behaviors in the tissue or system. We investigate this problem by applying a potent external migratory cue—electrical stimulation and electrotaxis—to primary mouse skin monolayers where we can tune cell–cell adhesion strength to modulate endogenous collectivity. Monolayers with high cell–cell adhesion showed strong natural coordination and resisted electrotactic control, with this conflict actively damaging the leading edge of the tissue. However, reducing preexisting coordination in the tissue by specifically inhibiting E-cadherin–dependent cell–cell adhesion, either by disrupting the formation of cell–cell junctions with E-cadherin–specific antibodies or rapidly dismantling E-cadherin junctions with calcium chelators, significantly improved controllability. Finally, we applied this paradigm of weakening existing coordination to improve control and demonstrate accelerated wound closure in vitro. These results are in keeping with those from diverse, noncellular systems and confirm that endogenous collectivity should be considered as a key quantitative design variable when optimizing external control of collective migration.

## Significance

As collective cellular migration is critical for multicellular processes such as healing, approaches to reprogram it are of great interest. However, little is known about what happens when external “commands” clash with natural collective behaviors in a tissue. We investigate this question using a bioelectric stimulus—electrotaxis—to externally program cell migration in large, cultured layers of primary mouse skin monolayers, demonstrating how strong endogenous cell coordination competes with external migration commands, reducing tissue responsiveness to the commands and even causing significant cellular damage. However, we show that specifically weakening natural cell coordination by disrupting E-cadherin–mediated cell–cell adhesion improves our ability to control collective cell migration. These results offer a general approach to externally controlling highly coordinated tissues.

Collective cell migration enables intricate, coordinated processes that are essential to multicellular life, spanning embryonic development, self-healing upon injury, and cancer invasion modes (1). Control of collective cell migration, therefore, would be a powerful tool for biology and bioengineering as such control would enable fundamentally new ways of regulating these key processes, such as enabling accelerated wound healing. Efficient and precise control over cell motility is becoming increasingly feasible with modern biotechnologies. Tunable chemical gradient generators can redirect chemotaxing cells (2, 3), optogenetics can allow dynamic control of cell contractility (4), micropatterned scaffolds can constrain and direct collective growth (5), and recent work in bioelectric interfaces has even demonstrated truly programmable control over directed cell migration in two dimensions (6, 7). However, despite advances in sophisticated tools, applying them to complex cellular collectives raises a fundamental problem: What happens when we command a tissue to perform a collective behavior that competes with its natural collective behaviors?

Paradoxically, those endogenous collective cell behaviors already present in tissues are both a boon and bane for attempts to control and program cell behavior. On the one hand, endogenous collective cell migration means the cells already have established mechanisms for coordinated, directional migration that external cues and control can leverage. For instance, cadherin-mediated cell–cell adhesions in tissues mechanically couple cells together and allow for long-range force transmission and coordinated motion. This coupling allows tissues to migrate collectively and directionally over large distances and maintain cohesion and organization far better than individual cells might (8, 9). On the other hand, imposing a new behavior over an existing collective behavior may generate conflicts. Tight cell coupling can create a “jammed state” or solid-like tissue where cells are so strongly attached and confined that they physically lack the fluidity to migrate as a group (10, 11). Strong coordination established via physical coupling can hinder cells from responding to signals for migration, as shown by the need for zebrafish and other embryos to weaken cell–cell junctions prior to gastrulation to ensure cells collectively migrate to necessary locations (12–14). Hence, how “susceptible” a collective system may be to external control likely depends on a tug-of-war between the resilience and strength of the natural collective processes and the potency of the applied stimulus.

Here, we specifically investigate the relationship and interplay between an applied, external command attempting to direct collective cell migration and the strength of the underlying collective behaviors already present in the tissue. We address two key questions. 1) How much does the strength of an endogenous collective migration behavior in a tissue limit our ability to control its collective cell migration? 2) How can we circumvent such limitations? To investigate these questions, we needed both a programmable perturbation capable of controlling collective migration and a physiologically relevant model system allowing for tunable “collectivity.” Here, we use collectivity to describe how strongly cells are coordinated with their neighbors during migration—highly collective cells exhibit strong, coordinated motion and vice versa. As a perturbation, we harnessed a bioelectric phenomenon called “electrotaxis”—directed cell migration in direct current (DC) electric fields—using our SCHEEPDOG bioreactor (6). Briefly, electrotaxis arises when endogenous, ionic fields form during healing or development (∼1 V/cm) and apply gentle electrophoretic or electrokinetic forces to receptors and structures in cell membranes, causing them to aggregate or change conformation to produce a front–rear polarity cue (15, 16). Components spanning phosphatidylinositol phosphates (PIPs), extracellular signal-regulated kinase (ERK), phosphatidylinositol 3-kinase (PI3K), phosphatase and tensin homolog (PTEN), and small guanosine triphosphate (GTP)ases have been implicated in the transduction process, while gap junctions appear to have an inconclusive role (8, 17–19). Crucially, electrotaxis may be one of the broadest and most conserved migratory cues, having been observed in vitro in over 20 cell types across multiple branches of the tree of life (20–22). As electrotaxis in vitro appears to globally stimulate all cells equally and still induce directional motion, it is distinct from more locally dependent cues such as chemotaxis and haptotaxis. However, as no other reported cue has as much versatility and programmability, electrotaxis is an ideal choice for a broadly applicable cellular control cue in this study.

To complement electrotaxis, we chose primary mouse skin for our model system as skin injuries were where the endogenous electrochemical fields that cause electrotaxis were first discovered (in vivo, the wound boundary is negative relative to the surrounding epidermis), and we and others have shown layers of keratinocytes to exhibit strong electrotaxis (6, 23–25). Critically, primary mouse keratinocytes have tunable collectivity in culture as the cadherin-mediated cell–cell adhesion strength in this system can be easily tuned by varying calcium levels in the media—with low-calcium media thought to mimic conditions in the basal layers of the epidermis with weak adhesions and high-calcium media akin to conditions in the uppermost layers of skin with strong adhesions (26–28).

Together, these experimental approaches allowed us to precisely explore how the ability to externally “steer” collective migration in a living tissue using a powerful bioelectric cue depends on the native collectivity of the underlying tissue. First, we quantify collective strength in cultured skin layers by measuring neighbor coordination of cellular motion [a standard metric for collective motion adapted from collective theory (29)] and then, validate that the collectivity can be tuned in our model system of mouse keratinocyte monolayers by calibrating junctional E-cadherin levels. Next, we demonstrate how applying the same electrical stimulation conditions to tissues with differing native collectivity results in radically different outputs, with weakly collective tissues precisely responding to our attempts to control their motion, while strongly collective tissues exhibited detrimental supracellular responses resulting in tissue collapse. We then prove that E-cadherin is responsible for these differences, ruling out any effects of calcium signaling per se. Finally, we leverage these findings to develop an approach that allows us to effectively control mature, strongly collective tissues, which we utilize to demonstrate that we can accelerate wound repair in vitro.

## Results

### Establishing Baseline Collective Migration of Primary Keratinocyte Layers.

To determine how natural collective cell behaviors compete with externally imposed control of collective behavior, we first need to establish baseline data of endogenous collective behavior in the absence of guidance cues. We used monolayers of mouse primary keratinocytes as a model system as their endogenous collective behavior is well characterized (24, 30), they have a strong electrotactic response (6), and their cell–cell adhesion levels can be easily tuned via calcium levels in the culture media (28, 31). Previous work has indicated that cell–cell adhesions via calcium-dependent proteins, E-cadherin adhesion being one of the best studied, are essential in interconnecting individual cells and maintaining coordination within the monolayers by coupling mechanical information via the cadherin–catenin–actin complex (32–35). Hence, we hypothesized that modulation of cell–cell adhesion levels via calcium control would allow us to tune the relative strength of collective couplings and collective migration in primary keratinocyte layers, giving us a precise and reproducible system to explore questions of collective control.

**Fig. 1.**
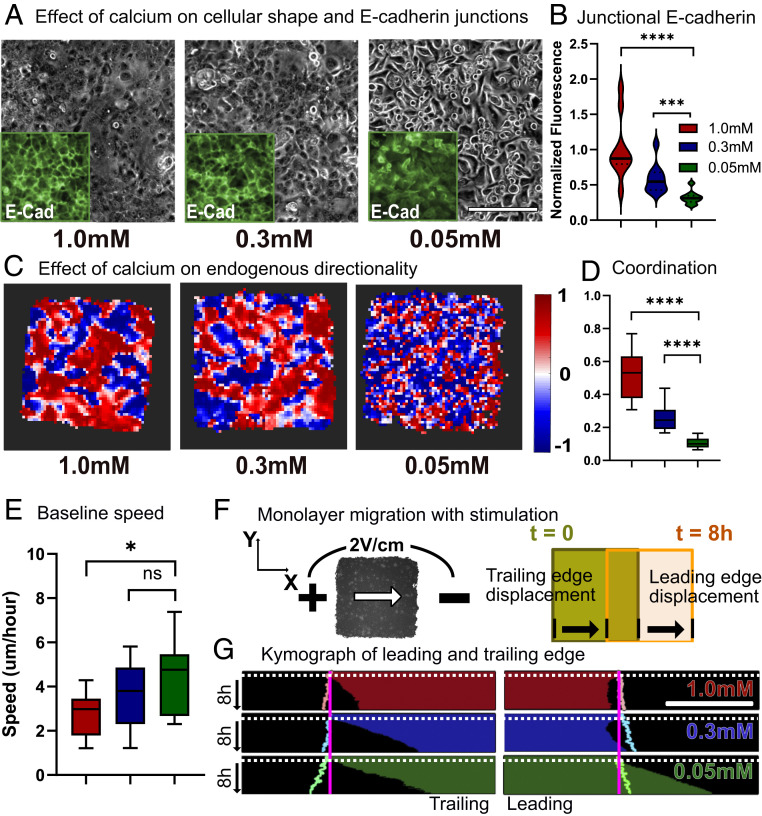
Baseline collective behavior of keratinocyte monolayers. Endogenous coordination increases with calcium-dependent cell–cell adhesion. (*A*) Phase and E-cadherin imaging for primary mouse keratinocyte monolayers cultured in high-calcium (1.0 mM), medium-calcium (0.3 mM), and low-calcium (0.05 mM) media for 14 h. Gray indicates the phase image; *Insets* (green) show immunofluorescence images of E-cadherin. (Scale bars: 200 μm.) (*B*) Distribution plot for normalized junctional E-cadherin immunofluorescence signal. (*C*) Horizontal directionality heat map. Each square corresponds to 40 to 45 μm of the monolayer. (*D*) Distribution plot for coordination values. The legend is identical to that in *B*. (*E*) Baseline migration speed. (*F*) Schematic for keratinocyte monolayer migration toward the cathode (leading and trailing edge displacement). (*G*) Leading and trailing edge displacement kymographs throughout 1 h of control (no stimulation) and 8 h of stimulation. Electrical stimulation starts at the white dotted line. Pastel outlines indicate the edge displacement of unstimulated monolayers of same calcium levels throughout 9 h. Error bars represent SD across tissues. *P* values are calculated using the unpaired nonparametric Mann–Whitney test. *n* = 15 for each condition. (Scale bar: 500 μm.) ns, not significant; *P < 0.05; ***P < 0.001; ****P < 0.0001.

To establish quantitative standards for collective strength in our keratinocyte model, we engineered arrays of identical $2-\times 2-{\text{mm}}^{2}$. keratinocyte tissues using tissue stenciling methods (6, 36). Tissue arrays were then cultured for 14 h in high-calcium (1.0 mM), medium-calcium (0.3 mM), or low-calcium (0.05 mM) conditions to allow junction formation (Fig. 1*A*). These calcium levels are standard conditions that span the physiological range based on phenotypes and marker expressions (28, 31, 37, 38). Using nuclei counting, we confirmed that density across conditions did not vary significantly (*SI Appendix*, Fig. S1). As E-cadherin is a major calcium-dependent adhesion protein, we used immunostaining to quantify and confirm the direct relationship between calcium level and E-cadherin recruitment to cell–cell junctions (Fig. 1 *A* and *B* and *SI Appendix*, Fig. S2). We generated collective motion data for each tissue by processing phase-contrast time-lapse movies captured using automated imaging with particle image velocimetry (PIV) to generate velocity vector fields for each time point (*Materials and Methods*). To ensure PIV fairly captured the range of conditions, we performed preliminary validations against analyses based on nuclear tracking and found no appreciable difference (*SI Appendix*, Fig. S3). The vector fields were then analyzed to visualize and quantify the strength of coordinated motion within a given tissue over time (*SI Appendix*, Fig. S4) (6, 23, 36). First, we calculated the directionality of cellular movements to visualize domains of coordinated migration within tissues. Directionality (Eq. **1**) is defined as the average of the cosine of θ, the angle between each PIV velocity vector and the horizontal *x* axis, while *N* is equal to the total number of velocity vectors in the frame. As the electric field command is in the horizontal direction (1 F), the directionality can also indicate how well aligned the cellular migration is with the field direction under stimulation. Directionality can vary between −1 (cell motion to the left; perfectly antiparallel with field) and 1 (cell motion to the right; perfectly parallel with field). Additionally, we quantified the collectivity by calculating the overall coordination within a tissue using the polarization order parameter (Eq. **2**) from collective theory, where $v_{i}$ indicates the ith velocity vector (29). A coordination value of one indicates perfect coordination and anistropy across the whole tissue, while zero indicates wholly isotropic motion:

$$Directionality=\frac{1}{N}\overset{N}{\underset{i=1}{\sum }}\cos\left(\theta \right)$$ [1]

$$Coordination=\|\frac{1}{N}\overset{N}{\underset{i=1}{\sum }}\frac{\vec{v_{i}}}{\left\|v_{i}\right\|}∥.$$ [2]

Our data (Fig. 1 *C* and *D*) clearly demonstrate that increasing calcium levels increases collectivity within the tissue. Both the general size of coordinated domains, represented by large zones of either red or blue in Fig. 1*C*, and the coordination parameter varied directly with calcium levels (Fig. 1*D*). Velocity correlation with nearest neighbors also increases in value with increased calcium levels (*SI Appendix*, Fig. S11). However, we also noted that increased coordination came at the cost of reduced average cell migration speed (Fig. 1*E* and Movie S1), suggesting that strong cell–cell adhesion impeded cellular motion, a common trade-off in collective motion (39). Notably, there is a clear shift in cell and tissue morphology across the different calcium levels, with high-calcium tissues visually exhibiting supracellular fluctuations and low-calcium tissues behaving far more like a dense collection of individualistic agents. Together with our data indicating that E-cadherin levels also vary directly with calcium and prior studies demonstrating a strong correlation between cadherin levels and coordination, these data validated our ability to tune endogenous collective strength in keratinocyte layers and to quantify and profile the natural collective motion of unstimulated tissues. With baselines established, we next investigated how collective strength regulates electrotactic susceptibility.

### Strong Collectivity Makes It More Difficult to Program Collective Cell Migration.

**Fig. 2.**
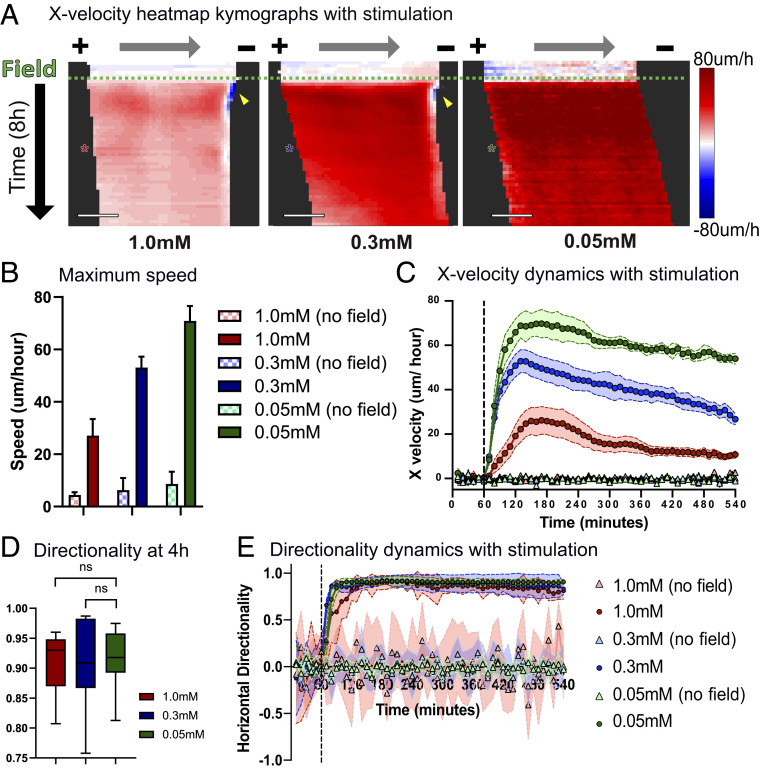
Increased coordination reduces monolayers’ responsivity to electrical stimulation. (*A*) X-velocity heat map kymograph for 1 h of control and 8 h of stimulation. Each square corresponds to 40 to 45 μm of the monolayer. Electrical stimulation starts at the green dashed line. Asterisks indicate 4 h into stimulation (10 min/row). (Scale bars: 500 μm.) (*B*) Maximum migration speed with and without electrical stimulation. (*C*) X velocity of migration throughout 1 h of control (no field) and 8 h of stimulation. Stimulation starts at the black dashed line. The legend is identical to that in *B*. (*D*) Horizontal directionality at 4 h into stimulation. (*E*) Horizontal directionality throughout 1 h of control and 8 h of stimulation. Stimulation starts at the black dashed line. Error bars represent SD across tissues. *P* values are calculated using the unpaired nonparametric Mann–Whitney test; *n* = 12 to 15 for each condition. ns, not significant.

Having related low calcium levels to weak collectivity and low junctional E-cadherin and high calcium levels to strong collectivity and high junctional E-cadherin, we next attempted to program and drive collective migration in these tissues using bioelectric stimulation. Here, we delivered a unidirectional electrotactic cue using a modified version of our SCHEEPDOG electro-bioreactor, which integrates a microfluidic bioreactor containing programmable electrodes around pregrown tissue arrays (*Materials and Methods*) (6). To mimic an endogenous field, we applied an electric field of 2 V/cm for 8 h across keratinocyte monolayers and observed as the monolayer migrated toward the negatively charged cathode (Fig. 1*F*). While all tissues responded strongly to the applied field, the nature of the response heavily depended on the collective strength of the tissue (Figs. 1 *G* and 2 and Movie S2).

Specifically, changes in collective strength impacted the spatiotemporal response of the tissue with respect to migration speed and directedness (Fig. 2*A*). While cells in all tissues increased their overall speed during electrotaxis as seen in previous work (6, 23, 36, 40–42), the relative increase in speed varied inversely with collective strength, with weakly collective monolayers migrating at almost twice the speed of strongly collective monolayers under the same electrical stimulation (Fig. 2 *B* and *C*). Faster motion in less strongly collective tissues was consistent with the baseline motility data without stimulation. Although the overall directedness of collective migration during electrotaxis was independent of collective strength, we noted that stronger collectives took longer to align than did weaker collectives, with the most strongly collective tissues taking 35 min longer to align than the other conditions (Fig. 2 *D* and *E*). This clearly demonstrates a competition between the endogenous collective behavior of a tissue and the imposed command, making more strongly collective tissues less responsive to bioelectric cues.

### Naive Collective Control Can Result in Catastrophic Damage to the Tissue.

**Fig. 3.**
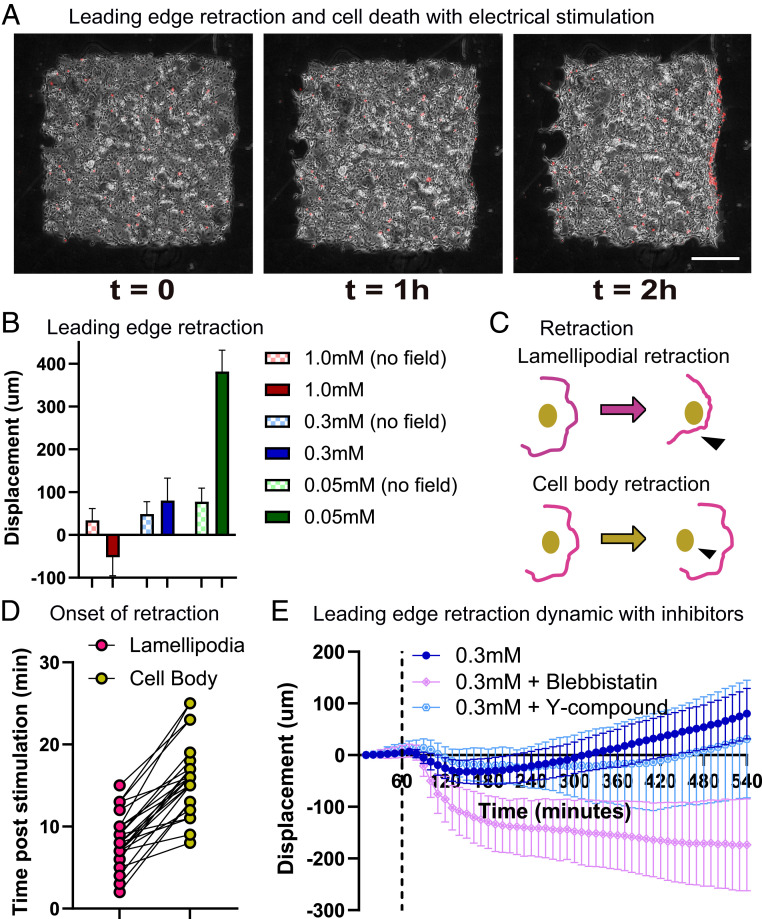
Leading edge retraction and cellular damage with stimulation in highly coordinated monolayers. (*A*) Phase (gray) and ethidium homodimer-1 (EthD-1) dye (red) images throughout 2 h of electrical stimulation of the medium-calcium monolayer. Yellow arrowheads indicate cell death and retraction at the leading edge. (Scale bar: 500 μm.) (*B*) Leading edge displacement after 8 h with and without electrical stimulation. (*C*) Schematic of lamellipodial retraction vs. cell body retraction with electrical stimulation. (*D*) Onset time of respective retraction postelectric stimulation (*n* = 24). (*E*) Leading edge displacement for medium calcium monolayers treated with blebbistatin (light pink) and Y-27632 (light blue) throughout 1 h of control and 8 h of stimulation. Stimulation starts at the black dashed line. Error bars represent SD across tissues; *n* = 10 for each condition.

Beyond differences in speed and response time, we observed a far more striking and detrimental phenotype; both our moderately and strongly collective tissues experienced powerful retraction and collapse of their leading edges, with the effect being more pronounced in strongly collective tissues (Figs. 1*G*, 2*A*, and 3*B*). Quantifying the dynamics of retraction revealed that it occurred within 15 min of electrical stimulation (Fig. 2*A* and *SI Appendix*, Fig. S5) in the moderate and strong collectives, while weakly collective tissues advanced with no apparent problems. Retraction also caused high cytotoxicity, a membrane damage marker (ethidium homodimer) (*Materials and Methods*) revealing strong and localized damage all along the retracting edge (Fig. 3*A*, *SI Appendix*, Fig. S6, and Movie S3). We quantified the overall effect of retraction by analyzing total leading edge displacement over 8 h of stimulation (Fig. 3*B*), where we see that strongly collective tissues experienced net negative forward motion, moderately collective tissues recovered some forward motion, and weakly collective tissues advanced nearly 4× over their unstimulated control case.

To better understand retraction, we analyzed higher frame-rate videos of the process and found that, in all cases, lamellipodial detachment preceded both cell blebbing and eventual retraction of the cell body (Fig. 3 *C* and *D* and Movie S3). Such retraction is strikingly reminiscent of tissue dewetting, a phenomenon in which cellular monolayers detach from the substrate and retract inward as E-cadherin junctions trigger myosin phosphorylation, increasing cortical tension within the monolayer (43, 44). That we do not observe retraction in single cells at any calcium level is also consistent with dewetting (Movie S4). As dewetting could be delayed by reducing contractility (44), we hypothesized that disrupting contractility in monolayers would also mitigate leading edge retraction. We used inhibitors to disrupt contractility in electrotaxing cell collectives by treating monolayers with either blebbistatin or Y-27632 dihydrochloride (Y-27632) at 20 μM for 1 h before electrical stimulation (42, 45) and maintaining inhibitor levels during perfusion and stimulation. However, both inhibitors failed to mitigate retraction—while Y-27632 had little effect, blebbistatin significantly worsened the phenotype (Fig. 3*E* and Movie S5). This suggests that simple contractility is unlikely to be the dominant driving force in leading edge retraction.

### Cell–Cell Adhesion Is Uniquely Responsible for Bioelectric Collective Migration Control.

**Fig. 4.**
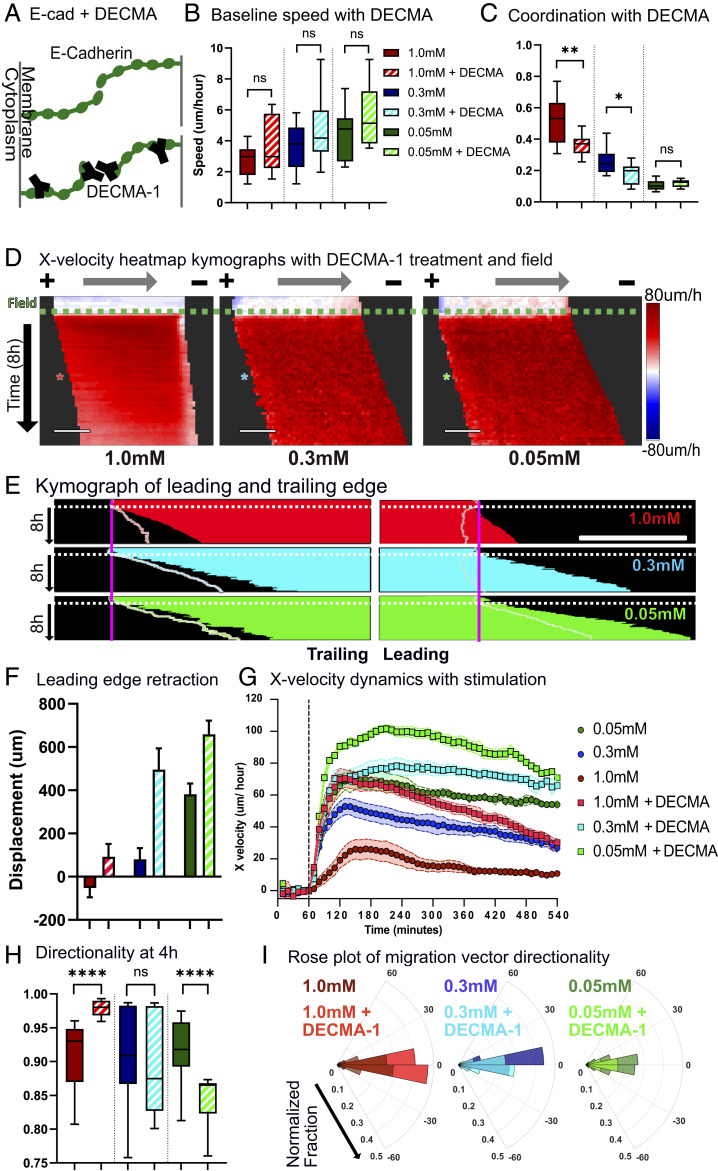
Disrupting E-cadherin junction formation with DECMA-1 reduces coordination and increases controllability. (*A*) Schematic of normal E-cadherin junction formation vs. with DECMA-1 disruption. (*B*) Baseline migration speed. (*C*) Coordination values. The legend is identical to that in *B*. (*D*) X-velocity heat map kymograph with DECMA-1 throughout 1 h of control and 8 h of stimulation. Each square corresponds to 40 to 45 μm of the monolayer. Electrical stimulation starts at the green dashed line. Asterisks indicate 4 h into electrical stimulation (10 min/row). (Scale bars: 500 μm.) (*E*) Leading and trailing edge displacement kymographs of DECMA-1–treated monolayers throughout 1 h of control and 8 h of stimulation. Electrical stimulation starts at the white dotted line. Pastel outlines indicate the edge of stimulated monolayers without DECMA-1 at same calcium level. (Scale bar: 500 μm.) (*F*) Leading edge displacement after 8 h of stimulation. The legend is identical to that in *B*. (*G*) X velocity throughout 1 h pf control and 8 h of stimulation with and without DECMA-1. Stimulation starts at the black dashed line. (*H*) Horizontal directionality at 4 h into stimulation. (*I*) Polar distribution plot of the velocity vector angle with respect to direction of the electrical field. The legend is identical to that in *B*. Error bars represent SD across tissues. *P* values are calculated using the unpaired nonparametric Mann–Whitney test; *n* = 12 to 15 for each condition. ns, not significant; *P < 0.05; **P < 0.01; ****P < 0.0001.

Based on our data showing correlation between collective strength and junctional E-cadherin, we hypothesized that E-cadherin–mediated cell–cell adhesion was a likely regulator of electrotactic control. To validate this and to rule out effects from calcium signaling (46–48), we treated tissues with a known blocking antibody against extracellular E-cadherin (CD324 (E-Cadherin) monoclonal antibody (DECMA-1)) (*Materials and Methods*) that specifically targeted and weakened cell–cell adhesion without altering calcium (Fig. 4*A*) (49, 50). While addition of the E-cadherin blocking antibody had little effect on migration speed, it had a pronounced effect on cell–cell coordination in the high- and moderate-calcium samples (Fig. 4 *B* and *C*).

Having down-regulated collective strength of tissues at all three calcium levels, we then tested how they responded to electrical stimulation. DECMA-1 treatment “rescued” forward motion by alleviating retraction in all calcium conditions (Fig. 2 *D* and *E* and Movie S6). Notably, all tissues experienced improvements to both forward motion (Fig. 4*F*) and average speed (Fig. 4*G*). That DECMA-1 treatment improved performance in even low-calcium tissues was notable as it implied that even the weak cell–cell adhesion still present in those tissues constrained the electrotactic response. Interestingly, while the overall speed and displacement of tissues were improved by blocking cell–cell adhesion, the accuracy or directionality of the collective migration response was more nuanced (Fig. 4*H*). DECMA-1 significantly increased the directionality in strongly collective monolayers while reducing directionality in weakly collective monolayers. To better relate this to accuracy or “spread,” we plotted polar histograms of the angles between cell velocity vectors and the electric field vector (Fig. 4*I*). Specifically, DECMA-1 decreased scattering perpendicular to the electrical field in electrotactic collective migration of strongly collective monolayers, while it increased scattering in weakly collective monolayers and made the control less precise. These data both suggested that overly strong native coordination, mediated specifically by E-cadherin in our experiments, can reduce controllability or cause adverse effects such as retraction.

While these results clearly demonstrate a key role for E-cadherin in regulating collectivity and control, skin is known to coexpress P-cadherin along with E-cadherin, which is known both to play a complex mechanobiological role and be able to rescue E-cadherin defects (51, 52). Hence, we also tested both specific disruption of extracellular P-cadherin using the blocking antibody P-cadherin monoclonal antibody (PCD-1) (53) and dual blockade of both P-cadherin/E-cadherin. However, while P-cadherin seems to play some minor role in electrotactic controllability, its role is neither as significant nor as conclusive as E-cadherin and, in certain cases, exacerbated undesirable phenotypes (*SI Appendix*, Fig. S7 and Movie S7). Usage of both DECMA-1 and PCD-1 together in an effort to down-regulate both E-cadherin and P-cadherin adhesion in medium calcium monolayers was also less effective than when only DECMA-1 was used (*SI Appendix*, Fig. S8). Together, all of these data suggest that overly strong native coordination, mediated specifically by E-cadherin here, can reduce controllability and even cause adverse mechanical effects such as retraction.

### Disassembly, Collective Transport, and Reassembly of a Tissue as a Control Strategy.

**Fig. 5.**
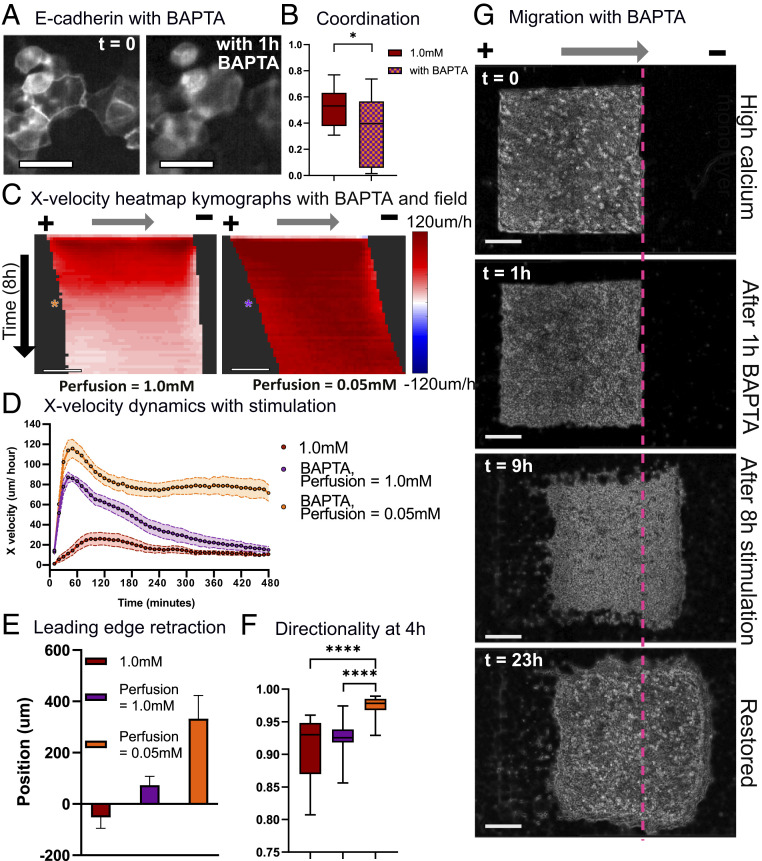
Controllability of highly coordinated monolayers can be easily and quickly rescued by acutely altering E-cadherin junctions. (*A*) GFP E-cadherin keratinocyte fluorescence images at *t* = 0 (*Left*) and with 1 h of BAPTA treatment (*Right*). (Scale bars: 20 μm.) (*B*) Coordination values for high-calcium monolayers and high-calcium monolayers treated for 1 h with 20 μM BAPTA. (*C*) X-velocity heat map kymograph for BAPTA-treated high-calcium monolayers stimulated in high- and low-calcium media. Asterisks indicate 4 h into electrical stimulation (10 min/row). (Scale bar: 500 μm.) (*D*) X velocity throughout 8 h of stimulation for high-calcium monolayers and high-calcium monolayers treated with BAPTA and stimulated in high- or low-calcium media. (*E*) Leading edge displacement of BAPTA-treated high-calcium monolayers after 8 h of stimulation in high- and low-calcium media. (*F*) Horizontal directionality at 4 h into stimulation. The legend is identical to that in *E*. (*G*) Phase image of high-calcium keratinocyte monolayers at *t* = 0 treated for 1 h with BAPTA (*t* = 1 h), electrically stimulated in low-calcium media for 8 h (*t* = 9 h), and restored in high-calcium media for 14 h (*t* = 23 h). Error bars represent SD across tissues. *P* values are calculated using the unpaired nonparametric Mann–Whitney test; *n* = 12 to 15 for each condition. (Scale bars = 500 μm.) *P < 0.05; ****P < 0.0001.

Knowing both that strong cell–cell adhesion can limit electrotactic control in skin and yet, that E-cadherin is essential for skin function and barrier formation, we sought to develop a more general stimulation strategy to allow us to transiently disrupt cell–cell junctions, use electrotaxis to reshape or move the more susceptible tissue, and then reassemble junctions when the tissue had reached its target location. While DECMA-1 treatment was effective at revealing the role of E-cadherin, it had three significant limitations to be used as a general approach: 1) Antibodies are expensive; 2) it is difficult to control how long it will block junctions; and 3) antibodies appear to have a difficult time penetrating already established strong cell–cell junctions (Fig. 4 *D*–*F* and *SI Appendix*, Fig. S8), thereby limiting their overall value in the very tissues we are trying to control more effectively. As an alternative, we tested brief exposure to 1,2-bis(o-aminophenoxy)ethane-$N,N,N^{\prime },N^{\prime }$-tetraacetic acid (BAPTA), an extracellular calcium-specific chelator (*Materials and Methods*), and examined how it disrupted E-cadherin junctions in preestablished tissues (54). Fluorescence imaging of green fluorescent protein (GFP) E-cadherin keratinocytes confirmed that 1 h of BAPTA treatment applied to tissues with strong E-cadherin junctions could transiently reduce junctional E-cadherin and reduce coordination (Fig. 5 *A* and *B*).

To test how rapid chelation affected the controllability of strongly collective monolayers, we treated monolayers with BAPTA for 1 h, washed out the chelator, and returned the monolayers to BAPTA-free, high-calcium media for electrical stimulation; 1 h of BAPTA treatment boosted controllability in strongly collective monolayers, with treated monolayers exhibiting both significantly increased migration speed and reduced leading edge retraction (Movie S8). However, these benefits were short lived, as speed and displacement drastically decreased over time (Fig. 5 *C* and *D*, orange) likely as cell–cell junctions eventually reengaged due to the high calcium concentration (*SI Appendix*, Fig. S10). To prevent the gradual restoration of junctions, we maintained tissues in low-calcium media after washing out BAPTA. These tuned tissues exhibited a nearly 5× increase in maximum speed, strong leading edge displacement, and high alignment with the field command (Fig. 5 *C*–*F*, purple).

Having confirmed that transient chelation could dramatically increase controllability, we then examined if we could restore the monolayer to its initial, highly coordinated state by removing the electrical field and returning disrupted monolayers to high-calcium media, allowing the calcium to reestablish junctions. E-cadherin fluorescence imaging shows that disrupted monolayers returned to high-calcium media overnight regained their contact with neighbors and reestablished strong E-cadherin junctions (*SI Appendix*, Fig. S10). Time-lapse imaging of the entire process—BAPTA treatment of strongly collective monolayers, migration in low-calcium media, and restoration in high-calcium media—demonstrates how a difficult to control tissue can be transformed to a more susceptible tissue, maneuvered to a desired location an arbitrary distance away, and then reassembled (Fig. 5*G* and Movie S9). In this case, while we do still note a thin zone of membrane damage at the initial leading edge (Movie S9, red band at the rightward edge), this no longer causes retraction, and the tissue instead surges forward as a cohesive unit.

### Accelerating Bioelectric Healing in Vitro by Manipulating the Strength of Cell–Cell Adhesion.

**Fig. 6.**
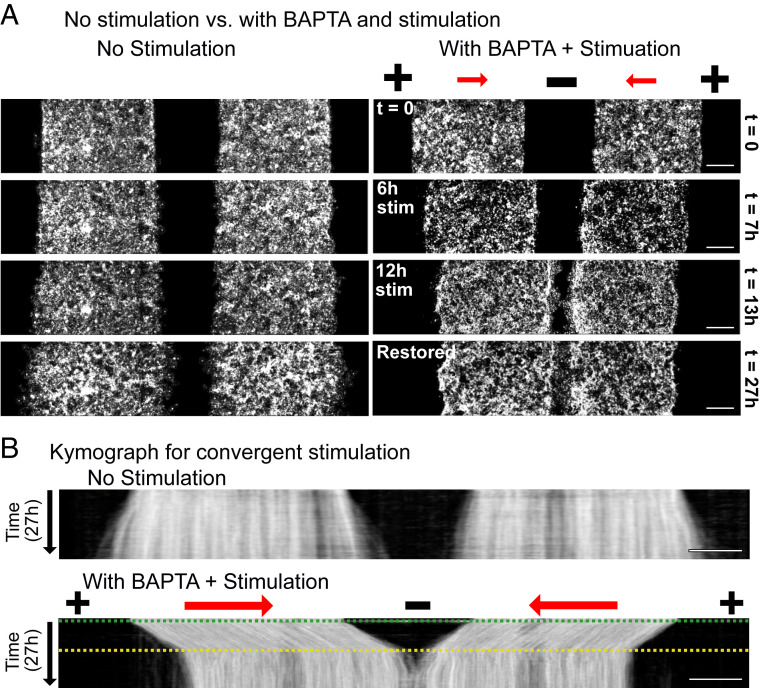
Accelerated in vitro wound healing. (*A*) Fluorescence images of unstimulated high-calcium monolayers (*Left*) and a high-calcium monolayer treated with BAPTA, convergently stimulated in low-calcium media for 12 h, and incubated in high-calcium media for 14 h (*Right*). (Scale bars: 500 μm.) (*B*) Kymographs of an unstimulated high-calcium monolayer (*Upper*) and a high-calcium monolayer treated with BAPTA, convergently stimulated in low-calcium media for 12 h, and incubated in high-calcium media for 14 h (*Lower*). The green dashed line indicates when the stimulation was switched on and media were changed to low-calcium media, and the yellow dashed line indicates when the stimulation was switched off and monolayers were returned to high-calcium media. (Scale bars: 500 μm.)

Combining pharmacological perturbations with bioelectric cues to improve tissue response suggests practical avenues to engineering the behavior of otherwise recalcitrant tissues for practical purposes. To test this, we created a wound gap across a strongly collective, high-calcium skin layer and reconfigured SCHEEPDOG to have a central negative electrode and peripheral positive anodes, generating an electric field that converged on the middle of the wound to drive each side of the tissue (*Materials and Methods*) (55). In this case, naïve stimulation would trigger a collapse or at best, no edge outgrowth (Figs. 2 and 3), but the disassembly/reassembly process described above should enable complete, expedited healing. Identical to the scheme described above, strongly collective monolayers were treated with BAPTA for 1 h, stimulated in low-calcium media, and restored in high-calcium media. The increase of wound closure rate for BAPTA + electrically stimulated tissues compared nonstimulated strongly collective monolayers is clearly visible in the time-lapse panels (Fig. 6 and Movie S10). Monolayers moved toward each other rapidly during the 12-h stimulation and successfully merged soon after they were returned to high-calcium media to restore their initial state. These data demonstrate both how controllability of tissues can be dynamically tuned and how such tuning can be used for practical effects—in this case, increasing the baseline wound closure rate by 2.5×.

## Discussion

“If you cannot join it, then beat it.” Our work demonstrates that the more strongly collective a given tissue is—determined here by cell–cell adhesion and native coordination levels—the more difficult it may be to externally program the behavior of that tissue as the command and the native behaviors compete with each other. A corollary to this is that, rather than synergizing with an existing collective behavior it can be beneficial to weaken, override, or “beat it.” In particular, our results demonstrate that we can better optimize the “controllability” of a cellular collective by both applying an appropriate external stimulus and modifying the internal, collective imperatives of the target system to mitigate the chance of conflict between imperatives.

Surprisingly, the consequences of ignoring the potential conflict between the command and natural imperative of a tissue can be quite drastic. While programmed electrotaxis of layers of weakly coupled primary mouse skin cells allowed for clean, large-scale control over tissue migration, the same electrical stimulation applied to strongly collective skin layers resulted in not only collapse of the leading edge of the tissue but also, considerable membrane damage in cells at the leading edge (Figs. 2 and 3). Some level of supracellular differences in behavior across an electrotaxing tissue—where the edges of a tissue seem less responsive than the bulk—has been noted in several prior electrotaxis studies in different models (6, 23, 36), but the collapse we see here has not been previously reported. Further, that inhibiting cell contractility (Fig. 3) worsened the problem suggests that collective contractility is not to blame for suboptimal electrotaxis and is consistent with prior data indicating that inhibiting myosin-mediated contractility does not abolish collective electrotaxis (42). As changes in cell-substrate dynamics, such as wetting–dewetting transitions, are also present in developmental processes such as morphogenesis, tumor formation, budding, and epidermal stratification, our results may suggest how electrically modulated retraction could be topical in such fields (56–58). Future work on cytoskeletal morphology and behavior at the leading edge of driven, collectively migrating tissues seems necessary to better clarify the role of the cytoskeleton in the collapse we observe.

However, we were able to successfully mitigate edge collapse and restore sustained directed motion across a whole tissue by specifically targeting E-cadherin to weaken cell–cell adhesion strength. Cell–cell adhesion, often regulated by E-cadherin, plays a critical role in collective cell migration as cell–cell junctions allow intimate coupling of physical forces and mechanical signaling across cells, enabling long-range coordination and the emergence of collective motion (59, 60). Our data linking reduced E-cadherin levels to weaker baseline coordination (Figs. 1 *B*–*D* and 4*C*), along with the results of specific inhibition of E-cadherin junctions (), support the concept that reducing E-cadherin adhesion tipped the balance in favor of electrotaxis, allowing the electrical cue to outcompete the now weaker internal collective prerogatives of the tissue. When the results are considered alongside prior findings where E-cadherin knockdown diminished electrotaxis in immortalized epithelial cells (8, 61), despite the complications in direct comparison due to differences in the cell type and baseline collective behaviors, the emerging story shows that while E-cadherin appears to be play a major role in regulating collective electrotaxis, either too little or too much cell–cell adhesion can detrimentally affect controllability. Hence, there appears to be a “Goldilocks” window for cell–cell adhesion strength and effective electrotactic control. Future work can further explore the effects of cell–cell interactions more broadly on electrotaxis, especially with respect to gap junctions, which facilitate ion and second messenger signaling.

This ability to independently tune internal collective strength and electrically stimulate a tissue externally suggested a solution to the problem of controlling strongly collective tissues: 1) transiently weaken internal collective coupling in a tissue, 2) bioelectrically drive the more controllable tissue to a target location or configuration, and 3) fully restore cell–cell coupling and tissue integrity at the new location. This approach ultimately allowed us to accelerate the collective healing process of a strongly collective, injured skin layer such that it healed at least twice as quickly as the control. Unexpectedly, we noted that electrotactic performance during this process of dynamically adjusting collective strength was improved, in terms of both speed and directionality, compared with tissues that began as weak collectives (Fig. 2 vs. Fig. 5). That we cannot only control collective cell behaviors but also, begin to optimize this control is exciting as there has been tremendous recent effort toward developing bioelectric wound dressings capable of improving healing in vivo (62–65).

Our results demonstrate the importance of native cell coordination and how it should be treated as an independent variable to be modified as needed when optimizing controllability of collective migration, such as with electrotaxis. As collectivity in cellular migration can be affected by factors such as cell density, geometric confinement, and proliferation, it is crucial to maintain such parameters constant as well as clarifying their effects for future research. With respect to the generality of the findings, we stress the importance of identifying factors beyond cell–cell adhesion that can be controlled to tune endogenous coordination in various other model systems, as well as establishing metrics to quantify collectivity. Similarly, electrotaxis is simply one possible stimulatory cue, albeit a potent and programmable cue, and alternative stimuli such as chemotactic gradients can also be explored in the role of “controller,” especially as endogenous collectivity has been shown to modulate chemotactic efficiency (12, 66). We hope our results and control paradigms presented here can help enable next-generation biointerfaces for clinical applications, a process that has been stalled despite promising results due to the difficulty of characterizing and observing the underlying mechanisms and lack of formal “design rules” for improved performance (25).

More broadly, our findings highlight underlying fundamental principles across collective systems and are in line with diverse examples of collective motion and control. For example, swarm theory predicts that overly strong collective coupling can reduce the responsiveness of the system to external perturbations, a finding consistent with experimental data across multiple systems (67). Panic in human groups can increase the strength and distance of correlated motion within the group, inhibiting the group’s ability to efficiently take advantage of exit cues and doorways during escape conditions (68). Similarly, swarms of locust nymphs have been shown to be more difficult to redirect when the natural structure of the swarm is denser and more aligned, and mathematical models of bee swarms showed that too strong attraction among individuals prevents scout bees from guiding the group (69–71). Finally, penguin huddles exhibit a natural clustering tendency, creating a jamming transition that would cause penguins on the outside of the group to die of exposure unless penguin clusters break symmetry and push their neighbors to transiently fluidize this jammed state and allow circulation from the outside in (72). In each of these examples, the underlying collective behaviors define the properties of the group, with stronger collectivity and coordination reducing the responsiveness and controllability of collectives. Given key similarities across collective systems, it is likely that there are many more guidelines from natural collective processes that we can take inspiration from to improve our ability to program tissues.

## Materials and Methods

Full materials and methods are available in *SI Appendix*, *Materials and Methods*. Primary mouse keratinocytes were seeded onto polydimethylsiloxane stencils on fibronectin-coated tissue culture plastic dishes and cultured in calcium-supplemented media. The electro-bioreactor was modeled after the SCHEEPDOG platform and directly assembled onto the tissue culture dish, delivering current from a Kiethly sourcemeter to the silver chloride electrode pairs. Field strength was maintained consistently at 2 V/cm using a custom MATLAB script. Cells were imaged using an automated Zeiss inverted fluorescence microscope. Image analysis and quantification were performed with FIJI (ImageJ) and MATLAB.

## Supplementary Material

Supplementary File

Supplementary File

Supplementary File

Supplementary File

Supplementary File

Supplementary File

Supplementary File

Supplementary File

Supplementary File

Supplementary File

Supplementary File

## Data Availability

All study data are included in the article and/or *SI Appendix*. Raw data/images and MATLAB scripts data have been deposited in Zenodo (https://zenodo.org/record/4730646#.YObrZehKiHs). High resolutions versions of the figures in this work are available on Zenodo (https://zenodo.org/record/4730646#.YObrZehKiHs) and GitHub (https://github.com/CohenLabPrinceton/PNAS-ShimEtAl2021).
