# Supplementary material for: Overriding native cell coordination enhances external programming of collective cell migration
Source: Proc Natl Acad Sci U S A. 2021 Jul 16;118(29):e2101352118. doi: 10.1073/pnas.2101352118 (PMC8307614; doi:10.1073/pnas.2101352118)
Supplement: Supplementary File [file pnas.2101352118.sapp.pdf]

Overriding native cell coordination enhances external programming of collective cell migration

Gawoon Shim, Danelle Devenport, Daniel J. Cohen

Supporting Information Text

Materials and Methods.

Cell maintenance. Primary keratinocytes and GFP E-cadherin keratinocytes were harvested from mice (courtesy of the Devenport Laboratory, Princeton University). Cells were cultured in E-medium supplemented with 15% serum and 50 µM calcium(1). All cells were maintained at 37 °C under 5% CO_2 and 95% relative humidity. Cells were split before 70% confluence and passage number was kept above 35 for all experiments. A 200mM Ca^{2+} stock (courtesy of the Devenport Laboratory, Princeton University) was diluted in E-Media to adjust the media calcium level as desired. Media was vortexed for 10 s after the addition of calcium and kept overnight at 4°C for even mixing.

Cell patterning. To seed monolayers in the stimulation zone and control their shapes and sizes, a silicone stencil of 250 µm thickness (Bisco HT-6240, Stockwell Elastomers) containing five $2 \times 2mm^2$ square microwells was cut and applied to the center of the culture substrate. The tissue culture plastic substrate was treated with fibronectin dissolved to 50 µg/mL in DI water, which was applied to the dish for 30 min at 37°C, then washed three times with DI water to provide a matrix for cellular adhesion. The microwells were spaced 4-6 mm from its neighbors to ensure each monolayer had access to sufficient nutrients. With the stencil in place, seeding solution of cells was prepared at a density of 2.5×10^6 cells, counted using Corning Cell Counter (Corning) and 2.5 µL of the cell solution was pipetted into the stencils. The cells were allowed to settle for 6 h with extra humidification media was added to the periphery of the tissue culture dish. Once the cells had adhered to the substrate, sufficient media supplemented with calcium was added to fill the dish. The stencils were removed 14 h after incubation for assembly.

Inhibitor assays. Monolayers were treated either 20µM blebbistatin (biogems) or Y-27632 (Selleckchem) for 1 h before stimulation after the patterning stencils were removed to prevent the inhibitors from being absorbed into the PDMS. Identical concentration of inhibitors was supplemented in the perfusion media to prevent the inhibitors from washing out during stimulation.

Live/Dead assay. Monolayers were treated with 1µM EthD-1 solution (LIVE/DEAD Viability/Cytotoxicity Kit, Invitrogen) for 1 h before stimulation after the patterning stencils were removed. Identical concentration of EthD-1 was supplemented in the perfusion media to prevent the reagent from washing out during stimulation.

Cell-cell adhesion disruption. To disrupt E-cadherin junction formation, cells were first seeded at desired density and allowed to settle for 6 h. Calcium supplemented medium containing DECMA-1 (CD324 (E-Cadherin) Monoclonal Antibody, eBioscience) at 50 µg/mL concentration was added to fill the dish for overnight incubation. After the 14 h incubation, DECMA-1 was washed out before the device was assembled for stimulation. Disruption of P-cadherin junctions was done using the same protocol with the P-cadherin specific antibody PCD-1 (P-cadherin Monoclonal Antibody, Thermo Fisher). For rapid E-cadherin junction disruption, high calcium monolayers were treated with 20µM BAPTA (Tetrasodium salt (cell impermeant), ThermoFisher) for 1 h. The chelator was washed out directly before the electrical stimulation.

Electrical stimulation. The uniaxial stimulation device assembly is a modified version of the SCHEEPDOG bioreactor from our prior work, and our methods are similar to those published previously(2). A Kiethly source meter (Kiethly 2450 Textronix) provided current to the silver chloride electrode pairs while an USB oscilloscope (Analog Discovery 2, Digilent Inc.) measured the voltage across the pair of recording electrodes using titanium wires (0.5 mm diameter, Alfa Aesar) as probes. A custom MATLAB script was written to use closed-loop feedback control to adjust the output current from the source meter to maintain the electrical field strength across the microfluidics channel constant at 2V/cm throughout the stimulation period. Device fabrication and instrumentation for convergent stimualtion were executed as published(3). Two $2 \times 2mm^2$ monolayers were spaced 1 mm apart with the cathode aligned over the center of the spacing. Anodes were aligned over the outer edges of monolayers to apply convergent electrical stimulation for 12 h, with the field strength kept constant at 2V/cm.

Microscopy. All images were acquired on an automated Zeiss (Observer Z1) inverted fluorescence microscope equipped with an XY motorized stage and controlled using Slidebook (Intelligent Imaging Innovations, 3i). The microscope was fully incubated at 37°C, and 5% CO2 was constantly bubbled into media reservoir during perfusion. Phase imaging was performed using a 5X/0.16 phase-contrast objective. Fluorescence imaging was performed using a metal halide lamp (xCite 120, EXFO). Immunofluorescence imaging for E-cadherin used a FITC filter set with a 20x/0.8 fluorescence objective and 500 ms exposure. GFP E-cadherin keratinocyte fluorescence imaging used a FITC filter set with a 20x/0.8 fluorescence objective and 200 ms exposure. Fluorescence imaging for EthD-1 used an RFP filter set with a 5X/0.16 phase-contrast objective and 100ms exposure time. Fluorescence imaging for nuclear and membrane dye used in convergent stimulation used a Cy5 filter set with a 5X/0.16 fluorescent objective and 350ms exposure time. Images were taken at 1 min or 10 min intervals as indicated in the text.

Immunofluorescence Staining. Cells were fixed in 4% paraformaldehyde (PFA) solution made by diluting a 16% PFA solution (Pierce 16% Formaldehyde, Thermo Scientific) in PBS. After 10 minutes, the cells were washed with PBS twice then permeabilized with 0.1% Triton X-100 (Sigma Aldrich) solution in PBS for 10 minutes. The cells were washed with PBS-Triton solution twice, then incubated with 1% Bovine Serum Albumin solution (Thermo Fisher) for 15 minutes for blocking for 1h. Primary antibody solution (DECMA-1, Thermo Fisher) diluted to 1:1000 in PBS was added and incubated for 1h. Cells were incubated with BSA solution again for 15 min and washed three times with PBS-Triton solution. The secondary antibody solution (Goat anti-Rabbit IgG (H+L) Alexa Fluor Plus 647, Invitrogen) diluted to 1:1000 in PBS was added and incubated for 30 min. Cells were washed 3 times in PBS-Triton solution, washed 3 times in PBS, and maintained in PBS at 4°C. All incubations and washes were done at room temperature.

Nuclear and Membrane Dye. For nuclear staining, Hoescht 33342 10mg/mL solution (Invitrogen) was diluted to 1:2000 in media for nuclear tracking assays and added to monolayers 1h previous to imaging. For nuclear tracking assays with electrical stimulation, the perfusion media was supplemented with an identical concentration of Hoescht 33342 to prevent the dye from washing out. Membrane dye (CellBright Red Cytoplasmic Membrane Dye, Biotium) was added to the seeding solution at 1:400 dilution for the convergent stimulation assay. Dye was added at the seeding stage as it could not infiltrate high calcium monolayers if added after the formation of E-cadherin junctions.

Image processing and analysis. All post-processing of monolayer microscopy images was performed using FIJI(4). Images were collected sequentially, stitched, and template matched to correct for stage drift prior to being analyzed.

Cell counting. Fluorescent nuclei images were thresholded using FIJI and segmented using the 'Watershed' function to distinguish individual nucleus in tightly packed regions. The total number of nuclei was counted using the 'Analyze Particles' function.

Particle Image Velocimetry. Velocity vector fields were generated using PIVLab, a MATLAB plugin performing FFT-based PIV(5). PIV analysis was performed over the entire monolayer and masked to exclude background noise. Iterative win-https://www.overleaf.com/project/5ffbbce2858b37a71c1b218cdow analysis was performed using first 128x128 pixel windows followed by 64x64 pixel windows, both with 50% step overlap. Vector validation excluded vectors beyond 5 standard deviations and replaced them with interpolated vectors. The vector fields were imported into MATLAB to calculate coordination, velocity, speed, and directionality. X-velocity heatmap kymographs were generated by calculating the average velocity at each horizontal point across the entire monolayer at each timepoint, then temporally stacking rows. Velocity correlations with 8 nearest neighbors were calculated for each vector within the monolayer using the following equation(6, 7) where N refers to the total number of vectors being analyzed, n is the number of neighboring objects (as we only look at nearest neighbors, this is 8) from the current object i, and j refers to 8 neighboring objects: $Correlation = \frac{1}{N}\sum_{i=1}^{N}\left(\frac{1}{n}\sum_{j=1}^{n}\left(\frac{\vec{v_i} \bullet \vec{v_j}}{|\vec{v_i}|\,|\vec{v_j}|}\right)\right)$

Nuclei tracking. Fluorescent nuclei image stacks of migrating monolayers were analyzed using the 'TrackMate' plugin in FIJI. Tracks were generated by detecting spots via Laplacian of the Gaussian filtering and linking the positions using Linear Motion Tracking. Velocity was extracted by importing the tracks into MATLAB.

Junctional E-cadherin quantification. Montage images of fixed, immunostained keratinocyte monolayers at each calcium level were captured using a 20X/0.8 objective and a FITC filter set, and then subjected to 2x2 summation binning to improve signal-to-noise. 10 junctions were chosen at random per calcium concentration as shown in the immunofluorescence images in Supplementary Figure 1. A rectangle of 20x100 pixel lengths (approximately 6x30 μm) was drawn across each junction, indicated in the figure with red lines, with the long edge of the rectangle perpendicular to the junction. The fluorescence intensity values were averaged across the short edge of the rectangle using 'Plot Profile' plugin in FIJI, an example intensity plot shown in Supplementary Figure 1. The normalized junctional E-cadherin values were then measured by calculating [Max. Signal – Min. Signal]/ [Min. Signal] of fluorescence intensity across each line plot.

Boundary edge displacement kymographs. Kymographs were produced using FIJI and MATLAB. For the kymographs, the X-position for the leading and trailing edge of the monolayers was averaged for each time point using a sum averaging algorithm, then stacked temporally. The kymographs from multiple monolayers were then averaged using MATLAB for each experimental condition. Leading edge displacement was calculated by measuring the distance between the initial averaged X-position of the monolayer before stimulation and the final averaged X-position post stimulation. For the converging stimulation assay, images were masked to reduce background noise and increased in intensity with MATLAB to compensate for the lack of dye in newly proliferated cells. Masks were created by Gaussian-blurring phase or fluorescence images and thresholding.

Retraction quantification. Cell body and lamellipodial retraction at the leading edges were hand-tracked to determine how much time had elapsed since the start of electrical stimulation until the onset of each condition using timelapse videos (1 min/ frame) of medium calcium monolayers stimulated at 2V/cm.

1 Number of nuclei per monolayer for varying calcium concentration

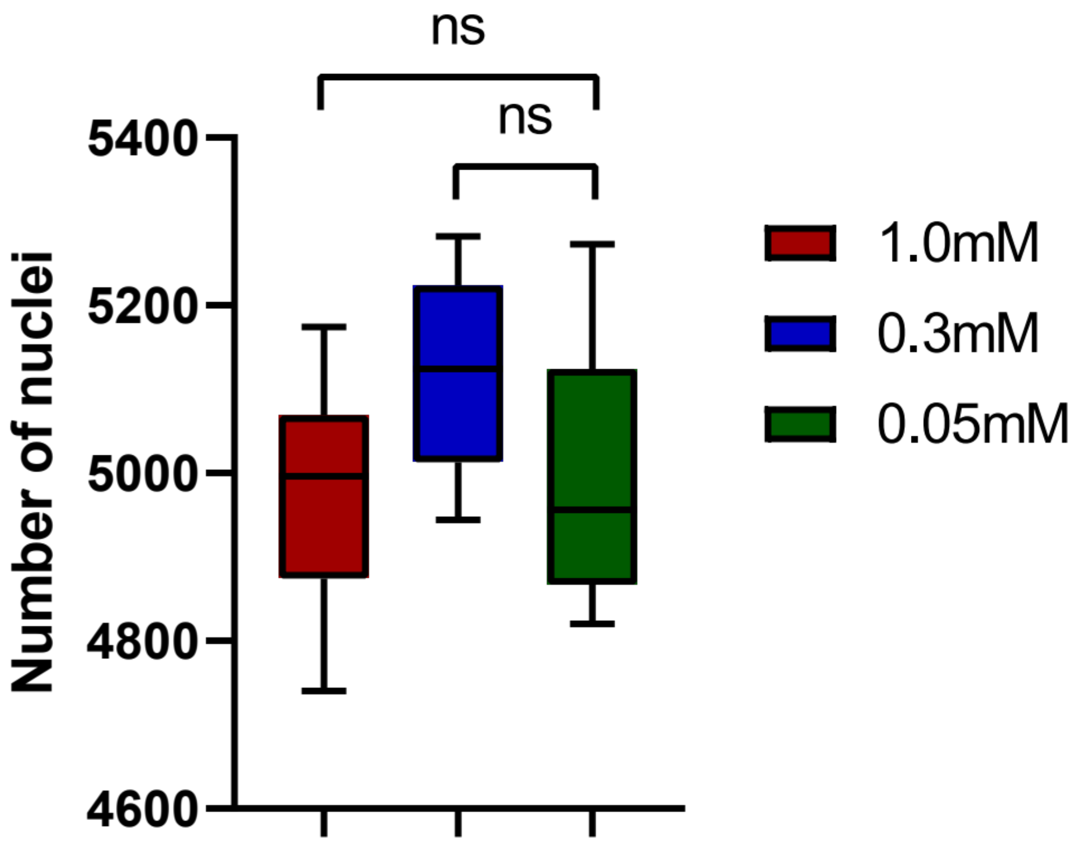

Fig. S1. Number of nuclei counted for monolayers incubated in high (1.0mM), medium (0.3mM), and low (0.05mM) calcium media for 14h.

2 E-cadherin junctions chosen for quantification and example of an averaged intensity plot across junction

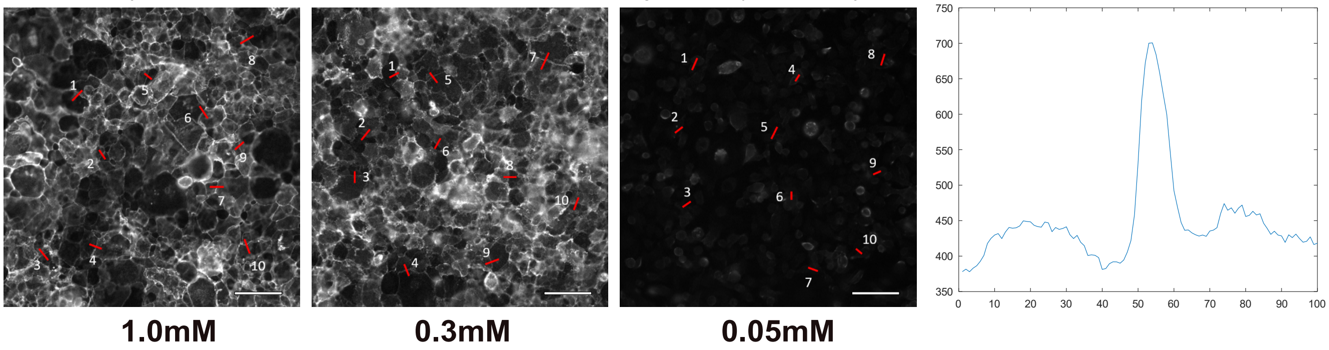

Fig. S2. Immunofluorescence imaging for E-cadherin with DECMA-1 for monolayers cultured in high (1.0mM), medium (0.3mM), and low (0.05mM) calcium media. Red lines indicate the cross-junctional lines across which the E-cadherin fluorescence intensity was quantified as shown in the line plot. Scale bar = $100\,\mu m$.

3 X-velocity measured using PIV Lab, MATLAB vs. nuclei tracking on Trackmate, FIJI

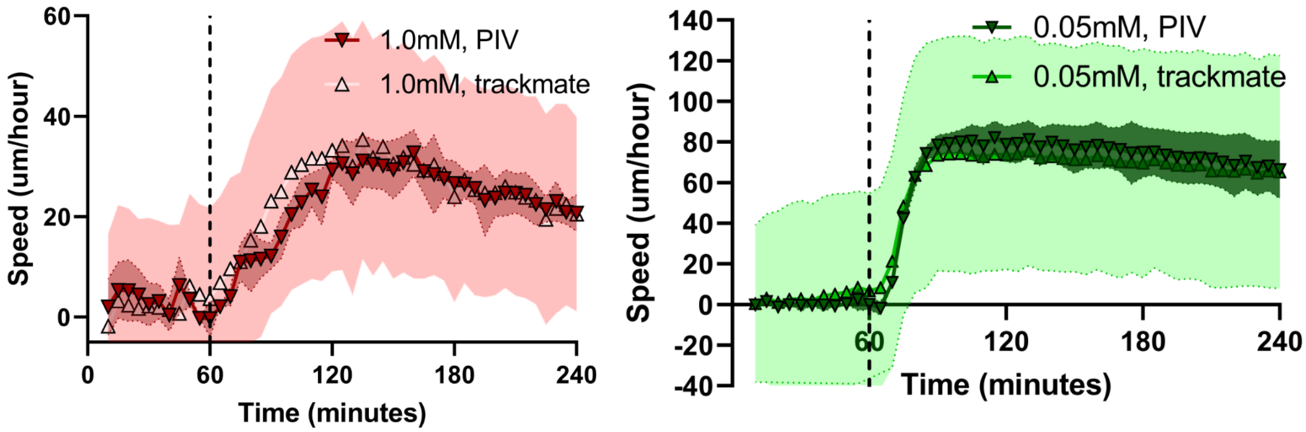

Fig. S3. Comparison of migration velocity measured using PIV (Matlab) and fluorescence nuclei tracking using Trackmate (FIJI) for high (1.0mM) and low (0.05mM) calcium media. n = 8-10 for each condition.

4 X-velocity heatmap kymographs without stimulation

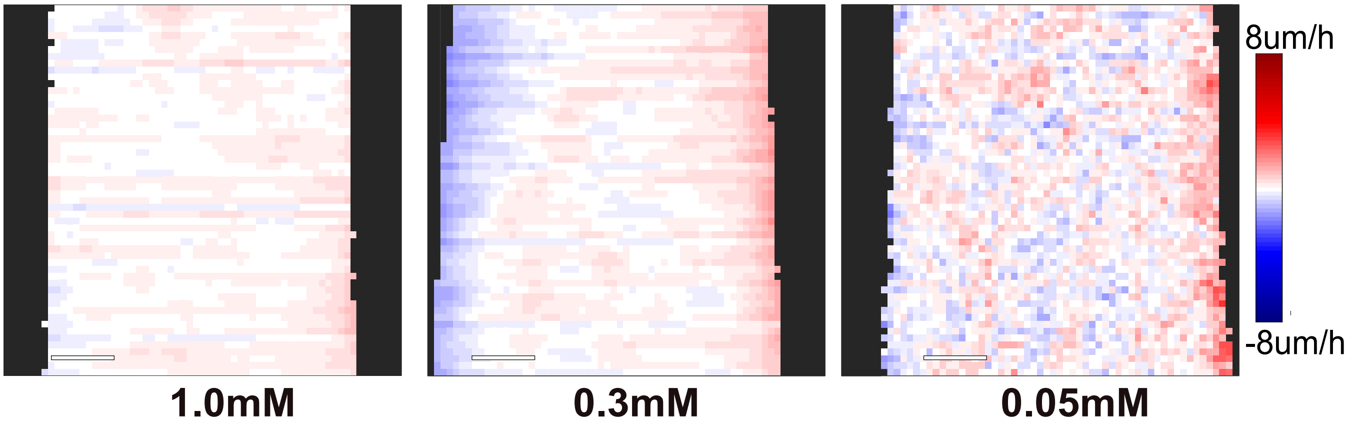

Fig. S4. X-velocity heatmap kymograph for varying calcium level without stimulation. n = 4-6, 3500-4000 tracks per monolayer. Scale bar = $500\,\mu m$.

5 X-velocity heatmap kymographs with stimulation, 0.3mM

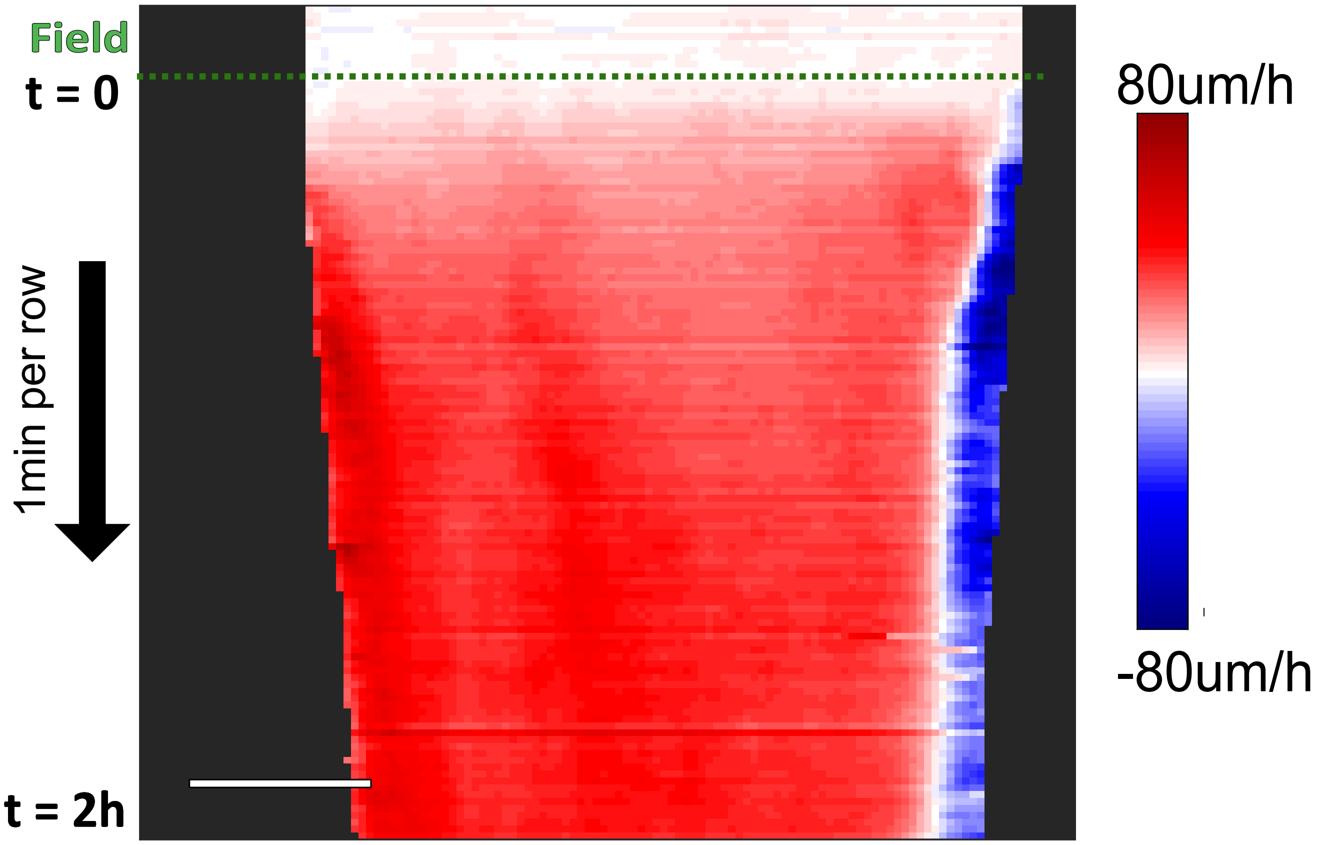

Fig. S5. X-velocity heatmap kymograph of electrotaxing medium calcium (0.3mM) monolayer, stimulated at 2V/cm. Stimulation starts at green dotted line. 1 min/ row. Scale bar = 500 µm.

6 EthD-1 fluorescence intensity plot with electrical stimulation

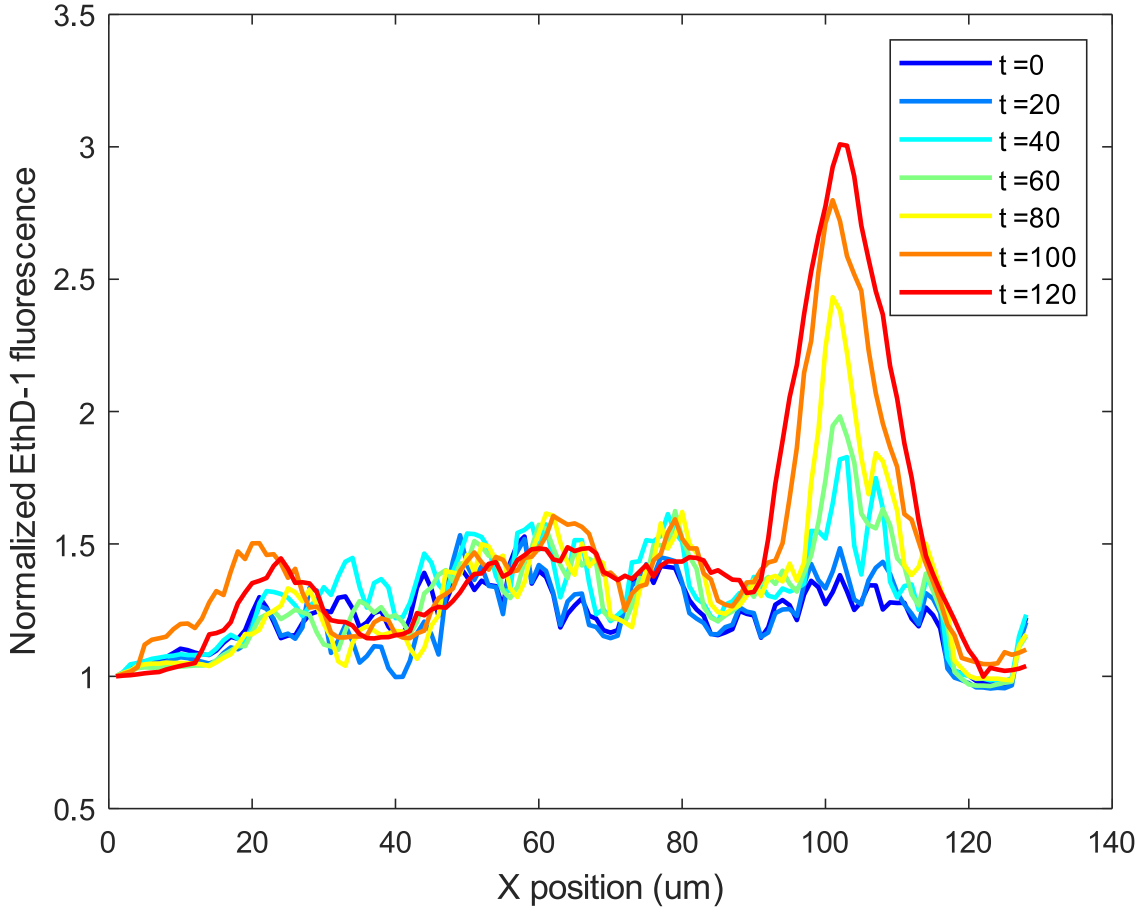

Fig. S6. Live-dead dye (EthD-1) fluorescence intensity across medium calcium monolayer (0.3mM) over 2 h with stimulation at 2V/cm. Monolayer migrates left to right, so the right edge is the leading edge. n = 5

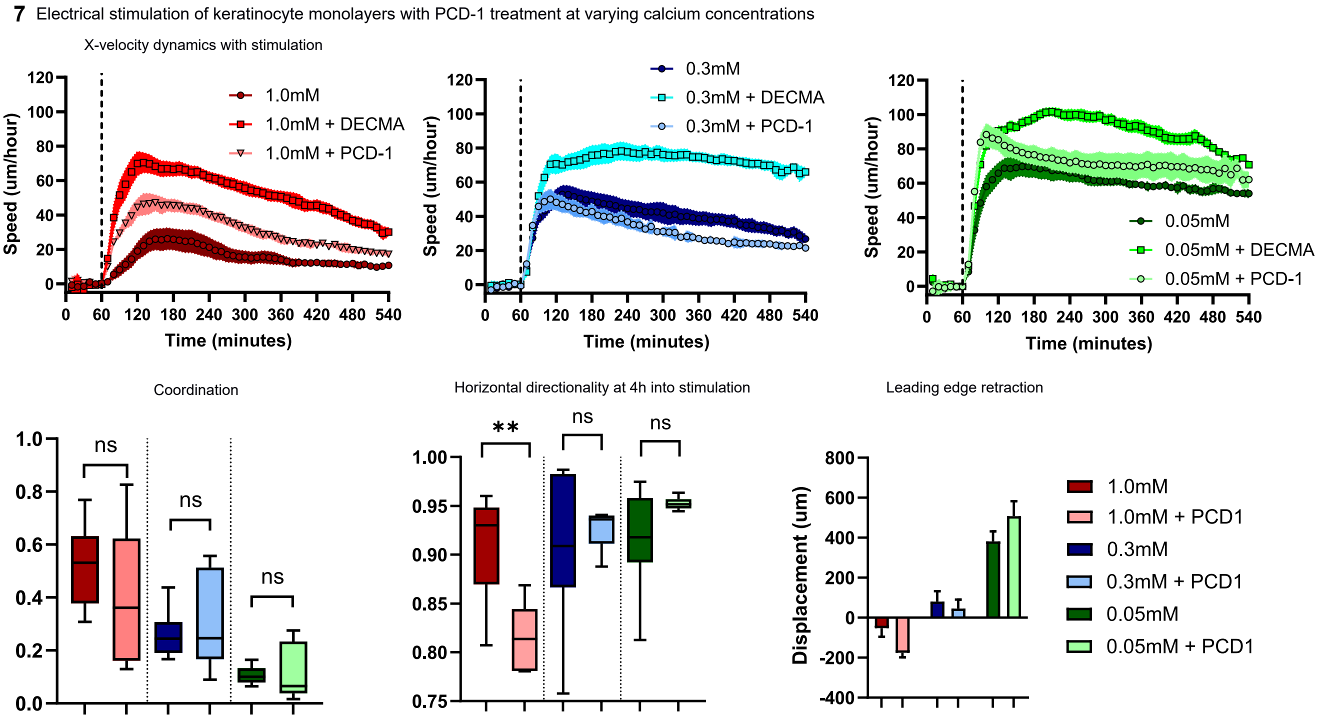

Fig. S7. X-velocity of migration throughout 1h control (no field) and 8h stimulation, coordination values, and horizontal directionality at 4h into stimulation for monolayers treated with PCD-1. n = 4-6 for each condition. * corresponds to $p < 0.05$, and ** to $p < 0.01$

8 X-velocity dynamics with stimulation for all tested conditions at 0.3mM

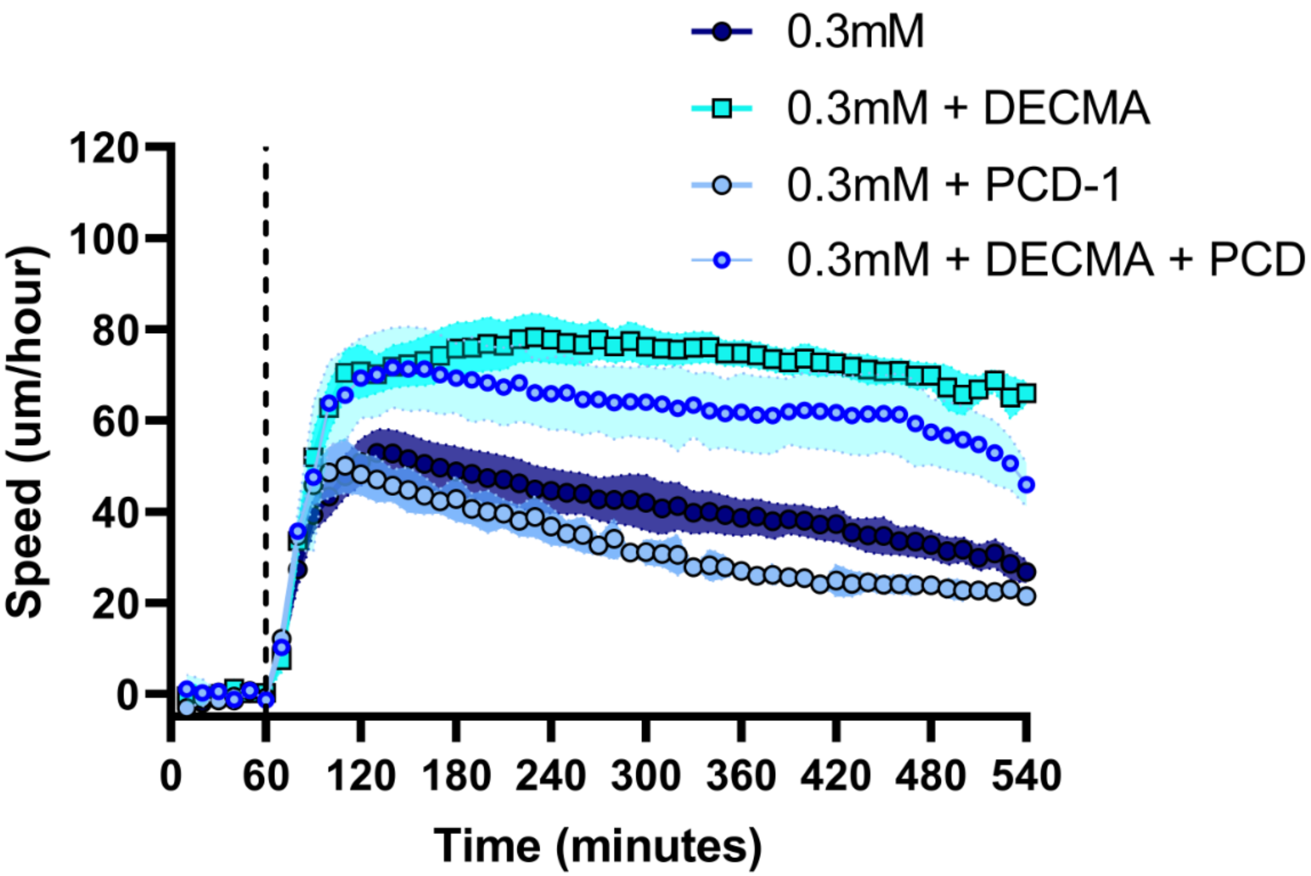

Fig. S8. X-velocity dynamics of medium calcium monolayers (0.3mM) without treatment, with DECMA-1 treatment, with PCD-1 treatment, and DECMA-1 + PCD-1 treatment during 1 h control and 8 h stimulation.

9 Coordination of monolayers formed in DECMA-1 added media vs incubated in DECMA-1 added media for 24h after monolayer formation in regular media

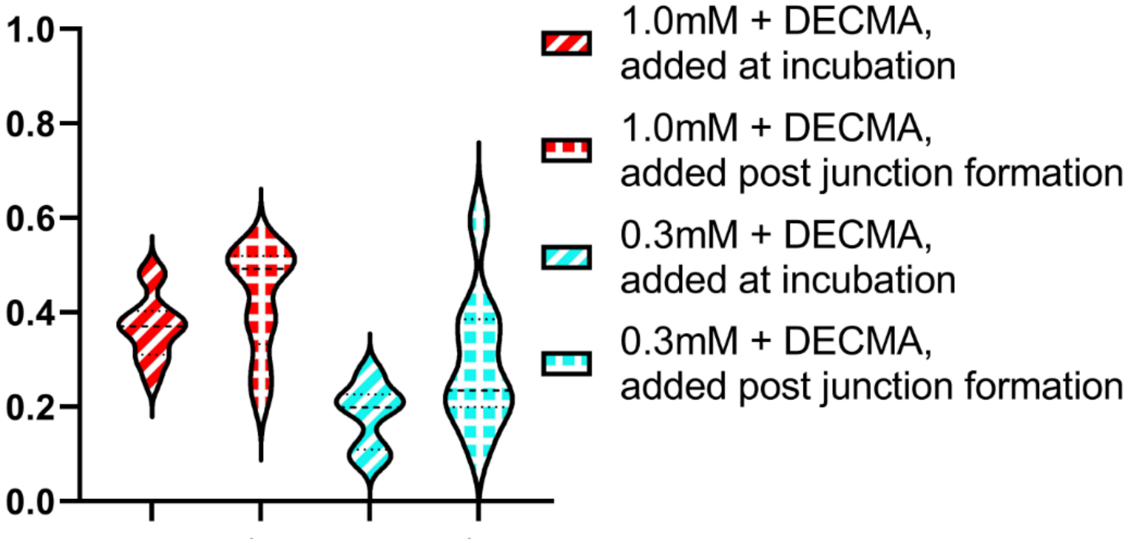

Fig. S9. Coordination values for monolayers formed with $50\,\mu g/mL$ added at the start of the 14 h incubation vs. monolayers treated with $50\,\mu g/mL$ DECMA-1 for 24 h after junctions had formed through the regular 14 h incubation.

10 Changes in junctional E-cadherin with BAPTA chelation and restoration

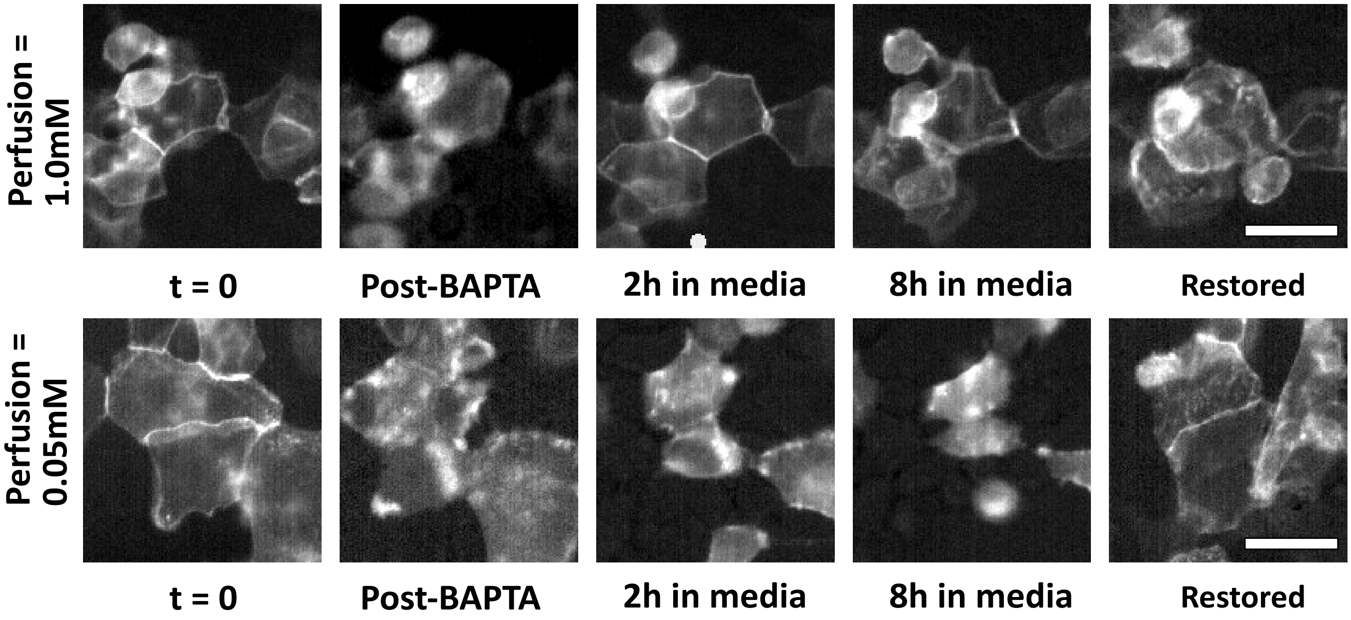

Fig. S10. E-cadherin fluorescence image of GFP E-cadherin cells. Top: Cells treated with $20\,\mu$M BAPTA, transferred to high calcium media, then incubated overnight in high calcium media. Image at t = 0, 1 h BAPTA-treatment (t = 1 h), 2 h in high calcium media (t = 3 h), 8 h in high calcium media (t = 9 h), and 14 h incubation in high calcium media (t = 23 h). Bottom: Cells treated with $20\,\mu$M BAPTA, transferred to low calcium media, then incubated overnight in high calcium media. Image at t = 0, 1 h BAPTA-treatment (t = 1 h), 2 h in low calcium media (t = 3 h), 8 h in low calcium media (t = 9 h), and 14 h incubation in high calcium media (t = 23 h). Scale bar = $20\,\mu\mathrm{m}$.

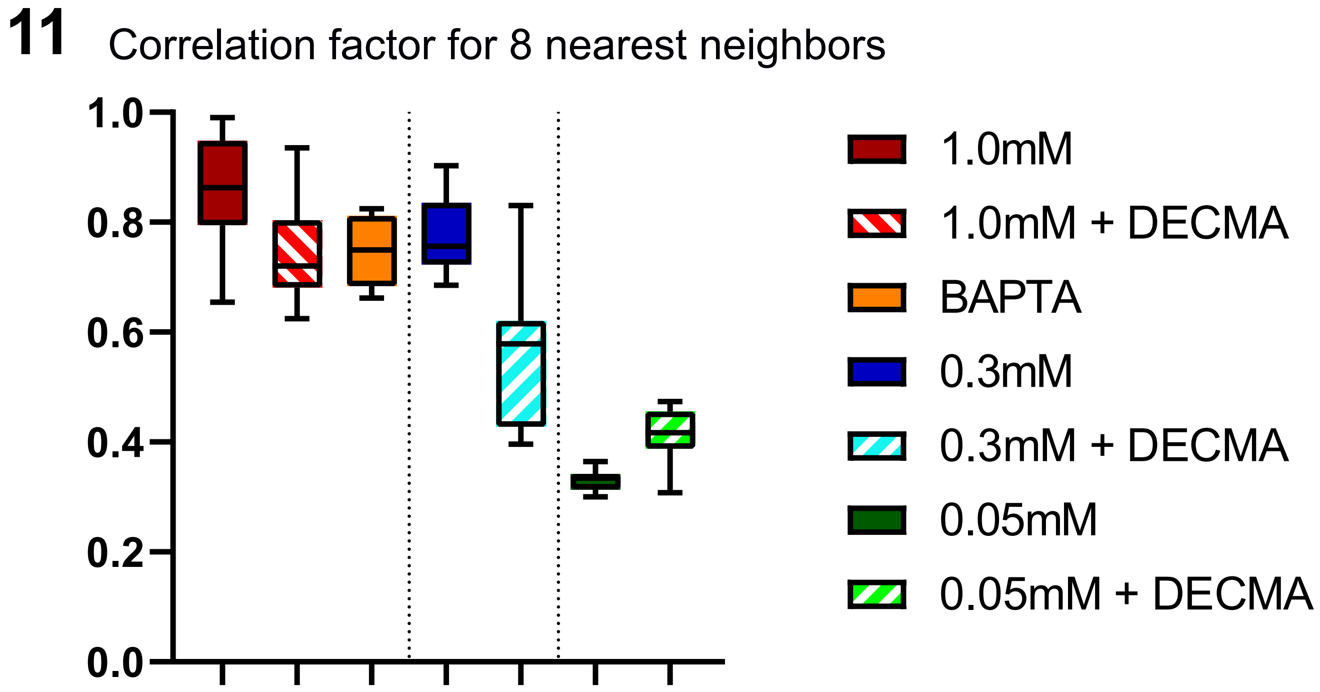

Fig. S11. Averaged velocity correlation values for 8 nearest neighbors calculated for each point in the monolayer for each experimental conditions (Methods).

Movie S1. Monolayers cultured cultured in high (1.0mM), medium (0.3mM), and low (0.05mM) calcium media. No field. 10 min/frame. Scale bar = 500 µm.

Movie S2. Electrotaxis of monolayers cultured and stimulated in high, medium, and low calcium media at 2V/cm. 1h control, 8 h stimulation. Electrical stimulation indicated with red circle. 10 min/frame. Scale bar = 500 µm.

Movie S3. Leading edge cell death using Live-Dead assay with EthD-1. Stimulation at 2V/cm with monolayer cultured and stimulated in medium calcium at 2V/cm. 1 min/frame. Scale bar = 500 µm.

Movie S4. Single cells cultured and stimulated in high and medium calcium media at 2V/cm. 10 min/frame. Scale bar = 100 µm.

Movie S5. Electrotaxis of monolayers cultured in medium calcium media, treated with 20µM blebbistatin and Y-27632 treatment, and stimulated at 2V/cm. 1h control, 8 h stimulation. Electrical stimulation indicated with red circle. 10 min/frame. Scale bar = 500 µm.

Movie S6. Electrotaxis of monolayers cultured in high, medium, and low calcium media, treated with 50 µg/mL **DECMA-1, and stimulated at 2V/cm. 1h control, 8 h stimulation. Electrical stimulation indicated with red circle. 10 min/frame. Scale bar =** 500 µm.

Movie S7. Electrotaxis of monolayers cultured in high, medium, and low calcium media, treated with 50 µg/mL **PCD-1, and stimulated at 2V/cm. 1h control, 8 h stimulation. Electrical stimulation indicated with red circle. 10 min/frame. Scale bar =** 500 µm.

Movie S8. Electrotaxis of monolayers cultured high calcium medium, treated in 20µM BAPTA for 1 h, and stimulated in high and low calcium media at 2V/cm. Electrical stimulation indicated with red circle. 10 min/frame. Scale bar = 500 µm.

Movie S9. Electrotaxis of monolayers cultured in high calcium media, treated with 20µM BAPTA for 1 h, stimulated in low calcium media for 8 h at 2V/cm, and restored without stimulation in high calcium media for 14 h. Red fluorescence is cell damage visualized with EthD-1. Electrical stimulation indicated with red circle. 10 min/frame. Scale bar = 500 µm.

Movie S10. Convergent electrotaxis of monolayers cultured in high calcium media, treated with 20µM BAPTA for 1 h, stimulated in low calcium media for 12 h at 2V/cm, and restored without stimulation in high calcium media for 14h. Electrical stimulation indicated with red circle. 10 min/frame. Scale bar = 500 µm.

References

1. KAU Gonzales, E Fuchs, Skin and its regenerative powers: an alliance between stem cells and their niche. Dev. cell **43**, 387–401 (2017).
2. TJ Zajdel, G Shim, L Wang, A Rossello-Martinez, DJ Cohen, Scheepdog: Programming electric cues to dynamically herd large-scale cell migration. Cell Syst. **10**, 506 – 514.e3 (2020).
3. TJ Zajdel, G Shim, DJ Cohen, Come together: bioelectric healing-on-a-chip. bioRxiv (2020).
4. J Schindelin, et al., Fiji: an open-source platform for biological-image analysis. Nat. methods **9**, 676–682 (2012).
5. W Thielicke, E Stamhuis, Pivlab–towards user-friendly, affordable and accurate digital particle image velocimetry in matlab. J. open research software **2** (2014).
6. TE Angelini, E Hannezo, X Trepat, JJ Fredberg, DA Weitz, Cell migration driven by cooperative substrate deformation patterns. Phys. review letters **104**, 168104 (2010).
7. H Haga, C Irahara, R Kobayashi, T Nakagaki, K Kawabata, Collective movement of epithelial cells on a collagen gel substrate. Biophys. journal **88**, 2250–2256 (2005).
